# Locus of Control, Self-Control, and Gender as Predictors of Internalizing and Externalizing Problems in Children and Adolescents in Northern Chile

**DOI:** 10.3389/fpsyg.2020.02015

**Published:** 2020-08-12

**Authors:** Jerome Flores, Alejandra Caqueo-Urízar, Cristián Ramírez, Giaela Arancio, Juan Pablo Cofré

**Affiliations:** ^1^Escuela de Psicología y Filosofía, Universidad de Tarapacá and Centro de Justicia Educacional, Arica, Chile; ^2^Instituto de Alta Investigación, Universidad de Tarapacá, Arica, Chile

**Keywords:** locus of control, self-control, internalizing and externalizing problems, infantile-juvenile, mental health

## Abstract

**Background:**

Both the control that people attribute to themselves over a situation (locus of control) and the control they attribute to themselves (self-control) have been proposed as aspects that can have an effect on internalizing problems in young people. There is little evidence of this relationship in the infantile-juvenile population in Latin America.

**Objective:**

To establish whether there is a significant predictive relationship of locus of control and self-control over internalizing and externalizing problems in the infantile-juvenile population, both at a general level and dimension-specific. These include depression, anxiety, social anxiety, somatic complaints, and post-traumatic stress.

**Methods:**

A cross-sectional-correlational study was carried out to establish if there was a possible predictive relationship in 3,664 schoolchildren of both primary (4th–6th grade) and secondary (7th–12th grade) in northern Chile, using the short version of the Nowicki-Strickland scale to measure locus of control, the Tangney scale to measure self-control, and the Child and Adolescent Evaluation System (SENA) to measure the dimensions of internalized problems.

**Hypotheses:**

(1) Greater self-control is associated with lower levels of internalizing and externalizing problems. (2) Higher external locus of control is associated with higher levels of internalizing and externalizing problems. (3) Self-control, locus of control, and gender can together significantly predict each of the internalizing and externalizing problems.

**Results:**

Evidence is found to support the first two hypotheses fully and partially support the third, since gender did not function as a predictor in all models.

**Conclusion:**

The results confirm previous international research in that both locus of control and self-control appear to have a significant influence on internalizing and externalizing problems. Implications for mental health promotion in this population are discussed.

## Introduction

Globally, it is estimated that 1 in 5 children and adolescents have mental health problems (World Health Organization, 2003, (2012)), affecting their current and future quality of life, academic achievement and social relations. Mental health is an especially relevant issue in Chile, and little research has been undertaken on the child and youth populations. In international comparisons, evidence has been found that children under 6 years of age have low mental health indicators (Rescorla et al., 2011, 2012). The last study that widely included this population was that of Vicente et al. (2012), which found more symptoms in children than in adolescents.

The classic categorization of mental health problems has been to consider them as either internalizing or externalizing (Achenbach and Edelbrock, 1981). Internalized mental health problems have been studied both together (White et al., 2013), but more often, they have been analyzed separately. These usually include depression, anxiety, social anxiety, somatization, post-traumatic stress, and obsessive-compulsive disorder (Balan et al., 2017; Boyraz et al., 2019). Externalized mental health problems include disruptive or inappropriate behaviors, such as Attention Deficit, Hyperactivity-Impulsivity, Anger Control Problems, Aggression, Defiant Behavior, and Antisocial Behavior (Jenson et al., 2011; Lewis et al., 2016; Van Heel et al., 2019; Bishop et al., 2020). The first two are sometimes considered to be specifically executive function problems (Re et al., 2015).

Analyzing psychosocial variables related to internalizing and externalizing problems would facilitate progress in their prevention and promotion of mental health. Ollendick and Grills (2016), in a review, suggest that locus of control and self-control should be measured simultaneously to better understand childhood anxiety. Depression has been found to be associated with locus of control and self-control in children (Garaigordobil et al., 2017), and both have presented a significant association with each other (Autry and Langenbach, 1985; Gwandure and Mayekiso, 2010).

Based on Social Learning Theory, Rotter (1954) developed the concept of locus of control of reinforcement, the way in which an expectation can be strengthened is central: “The effects of reward or reinforcement on preceding behavior depends in part on whether the person perceives the reward as contingent on his own behavior or independent of it” (Rotter, 1966, p. 1). External locus of control is when these effects are “typically perceived as the result of luck, chance, fate, as under the control of powerful others, or as unpredictable because of the great complexity of the forces surrounding him” (Rotter, 1966, p. 1). Conversely, a person is considered to have an internal locus of control, when they perceive that there is a cause-and-effect relationship based on their own behavior (Rotter, 1966).

The control that children and adolescents perceive over what happens to them can influence their emotional problems (Levin, 1992). Locus of control is affected by context, as reflected in a study of institutionalized children, who tended to have a higher external locus of control compared to those living with their families (Król et al., 2019). There is also the possibility that internalizing problems can be influenced by socio-economic factors including area of residence (McDermott et al., 2017).

Locus of Control has been found to be linked to mental health (Groth et al., 2019; Kesavayuth et al., 2019) and may predict suicide risk (Loftis et al., 2019) and discipline problems (Tony, 2003). Adolescents with a high external locus of control score tended to have more emotional problems when under high levels of stress (Huebner et al., 2001; Aspelmeier et al., 2012). Both their direct relationship to depression and anxiety and their possible mediating effects have been reported (Di Pentima et al., 2019). The effect of social stress on locus of control has also been considered (Millman et al., 2017). A longitudinal study found an association between an increased external locus of control and depression (Sullivan et al., 2017). Also, locus of control was found to be a significant predictor of externalizing problems in adolescents (Liu et al., 2000). When children perceive that they have little control over events, they are more likely to develop externalizing problems (Jackson et al., 2000).

“Self-control is the exertion of control over the self by the self. That is, self-control occurs when a person (or other organism) attempts to change the way he or she would otherwise think, feel, or behave” (Muraven and Baumeister, 2000, p. 247). Nigg (2017) proposes that self-control is an essential aspect of the voluntary process of self-regulation, being also key to the understanding of psychological problems. Self-control has also been proposed as a sub-domain of social skills (Gresham and Elliot, 1990). Students who have high self-control would get better grades, have higher self-esteem, as well as better interpersonal relationships, fewer psychological and emotional problems (Tangney et al., 2004). A negative relationship has been found between self-control and internalizing problems (Nie et al., 2014), particularly with depression (Jun and Choi, 2013) and anxiety (Sánchez-Aguilar et al., 2019). The possible mediating role of self-control between perceived stress and life satisfaction has also been considered (Zheng et al., 2019). It has also been proposed that self-control is important in relation to externalizing problems (Franken et al., 2015; Wills et al., 2016). In comparisons of adolescents and adults it has been shown that the former have less self-control (Oliva et al., 2019). Self-control is acquired from early childhood, is adaptive, and considered a protective factor against the onset of different psychopathologies and should be taken into consideration when developing public policies (Moffitt et al., 2011).

Gender differences have been found in the distribution of mental health problems in this population. It is more common for females to have internalizing problems (Chaplin and Aldao, 2013; Schäfer et al., 2017), conversely, males present with more externalizing problems (Navarro-Pardo et al., 2012; Campos et al., 2014).

The purpose of this study was to establish the relationship between locus of control, self-control, and internalizing problems in school children from the fourth year of primary school to the last grade of secondary education in northern Chile.

Most of the research in the areas mentioned above, focused on populations over 12 years of age, so considering a broader age range would be beneficial to the topic. In this research, the age range is 8–18 years old. There are no other studies in Chile that consider all these variables in the child and adolescent population, and can thence be used to inform mental health programs and public policies, and thus, contribute to improvements in the quality of life for young people.

We had three hypotheses:

(1) Greater self-control is associated with lower levels of internalizing and externalizing problems.
(2) Higher external locus of control is associated with higher levels of both internalizing and externalizing problems.
(3) Self-control, locus of control and gender can together significantly predict each of the internalizing and externalizing problems under consideration.

## Materials and Methods

### Methodology

Our study design was transactional-correlational as all variables were measured at a single time point (Hernández et al., 2010) and comprised approximately 10% of the school enrolment for the entire region.

### Participants

The total sample comprised 3,664 students, in two different age groups and used two different versions of the SENA (Child and Adolescent Evaluation System) instrument adapted to be age appropriate. Both groups included public, government-subsidized and private schools.

The primary school group comprised 1,387 students between the fourth and sixth year of primary education from different schools in Arica. Boys comprised 46% and girls 53.6%, and ranged in age from 8 to 11 years old (mean 10 years; standard deviation was 0.83 years). Just over half (52.1%) declared themselves to be Latin American, 26% Aymara, 6.4% Mapuche and the remainder as belonging to other ethnic groups.

The secondary school group included 2,277 students between the seventh and last year of primary education (49.6% male, 50.4% female). The age range was 12–18 years (mean 14.4; standard deviation was 1.8 years). Again just over half (56.1%) identified as Latin American, 25.7% Aymara, 4.4% Mapuche and the rest as belonging to other ethnic groups. Table 1 shows sociodemographic characteristics of participants at baseline.

**TABLE 1:** Sociodemographic characteristics of participants at baseline.

	***n***	**%**
**Gender**		
Female	1891	51, 6
Male	1773	48, 4
**Grade**		
4	536	38, 6
5	468	33, 7
6	383	27, 6
7	471	20, 7
8	460	20, 2
9	407	17, 9
10	358	15, 7
11	324	14, 2
12	257	11, 3
**Ethnicity**		
Aymara	945	25, 8
Quechua,	38	1, 0
Mapuche	189	5, 2
Afro-descendant	109	3, 0
Latin American	1999	54, 6
Other	384	10, 5

The automated electronic correction of the instruments used allowed for up to 10% of unanswered questions, so those cases that exceeded that percentage were immediately discarded from further analyses. The imputation of missing values was used, which did not exceed 1% of the answers to each item. Missing values were imputed by the mean. Cases with an inconsistency score ≥ 1.6 were disgarded as recommended by the SENA authors, As a result, a total of 120 cases were omitted from the final sample.

### Instruments

#### Child and Adolescent Evaluation System (SENA) (Fernández-Pinto et al., 2015)

This instrument was developed to detect a wide range of emotional and behavioral problems in the 3–18 age range. It was constructed and validated entirely in Spanish. Only internalizing and externalizing problem scales were considered in this research:

*Internalizing problems:* Depression, Anxiety, Social anxiety, Somatic complaints, Post-traumatic symptomatology, and Obsession-compulsion.

*Externalizing problems:* Attention problems, Hyperactivity-impulsivity, Anger control problems, Aggression, Defiant behavior, and Antisocial Behavior.

It is a 5-point Likert scale graded from “never or almost never” to “always or almost always.” The total of each dimension is the average of the answers that constitute it, which can vary between 1 and 5 in a continuous way. It has an Inconsistency control scale.

The primary school version (8–12 years) had 10 scales to measure mental health: 48 items measuring internalizing problems and 39 items measuring externalizing problems. The secondary school version had 12 scales to measure mental health: 58 items measure internalizing problems and 46 measure externalizing problems. Each of the dimensions varied between 3 and 14 items, with an average of 8 per scale. Some examples of the questions are: “I feel that no one cares what I do” (Depression); “I get anxious or overwhelmed by my problems” (Anxiety); “I get nervous when there are many people around” (Social Anxiety); “I get unpleasant images of things that have happened to me.” (Post-traumatic stress). A higher score indicated greater problems for each scale.

The present investigation used the self-report versions of primary 8–12 years and secondary 12–18 years. Recently, Sánchez-Sánchez et al. (2016) found that the reliability of their subscales is above 0.7 in Spain. In the present investigation, all scales had reliability above 0.7. SENA also includes demographic questions on age, gender and school grade.

#### The Short Version of the Child Internal–External Locus of Control Scale (CNSIE) (Nowicki and Strickland, 1973)

Developed from the full 40-item version, specifically for children and adolescents. It has one dimension of external locus of control with a YES/NO response type for each question. There are different versions of this instrument, but in the present study the 12-item version was used. Some items are reversed scored. A higher score indicates a higher external locus of control. Criteria validity and reliability test-retest have been found to be adequate (Nowicki, 1976; Nowicki and Duke, 1983; Nowicki et al., 2018). The Guttman Split-half coefficient in the present investigation was 0.55. A back-translation procedure was used to ensure compatibility.

#### Brief Scale of Self-Control (BSCS) (Tangney et al., 2004)

This is a 13-item Likert response scale, where 1 is “don’t like it at all,” and 5 is “like it a lot.” It was developed from the full 36-item scale. A higher score indicates greater self-control. Its reliability is 0.85, and it is one-dimensional. An example of an item is “I refuse things that are bad for me.” The BSCS has been used in more than 50 studies and has good evidence of reliability and validity (Lindner et al., 2015). Some items are reverse scored for analysis. A higher score indicates greater self-control. The reliability in the present investigation was 0.68. The Spanish version was provided by the author of the scale.

### Procedure

(1) Approval of the Ethics Committee of the University of Tarapacá. This study formed part of a larger project of the Educational Justice Center.
(2) Forty-two educational establishments in the city of Arica were invited to participate in the study. A total of 69% (29) of schools agreed to participate.
(3) Informed parental and student consent was obtained.
(4) Assessment was carried out within the school classes. At least two trained interviewers were present to answer questions together with the class teachers. The questionnaires were given in the order: CNSIE, BSCS and finally SENA. Administration was approximately 45 min.

### Data Analysis

The demographic descriptions of each sample were presented, and then the basic statistics of the dimensions of the SENA. Correlations between locus of control, self-control, internalizing and externalizing problems in each sub-dimension of the SENA were then presented for each group. Examination of results showed little deviation from the norm. Given that the variables did not seem to be significantly skewed or have issues with kurtosis, Pearson correlations were used as the variables could take continuous values between 1 and 5. Multiple linear regressions for gender and both psychosocial variables on each of the internalizing and externalizing problems was then displayed. SPSS version 22 program was used. A stepwise regression was undertaken.

## Results

### Primary School Sample

The values of means, standard deviations, asymmetry, kurtosis, and the possible range of the dimensions of internalizing and externalizing problems of the SENA, locus of control, and self-control in the primary school sample are shown in Table 2. It can be seen that asymmetry and kurtosis for almost all are in acceptable ranges of ±2 indicating that the variables are normally distributed (George and Mallery, 2010). All were in the range proposed by Ryu (2011) for acceptable normal distributions.

**TABLE 2:** Descriptive statistics internalized and externalized problems SENA primary.

	**Minimum**	**Maximum**	***M***	***DS***	**Asymmetry**	**Kurtosis**
Depression	1,00	5,00	1,96	0,76	1,191	1,255
Anxiety	1,00	5,00	2,23	0,75	0,666	0,251
Social anxiety	1,00	5,00	2,45	0,83	0,416	−0,269
Somatic complaints	1,00	5,00	2,18	0,74	0,645	0,118
Post-traumatic stress	1,00	5,00	2,22	0,77	0,656	0,024
Attention problems	1,00	5,00	2,26	0,75	0,662	0,363
Hyperactivity- impulsivity	1,00	5,00	1,92	0,69	1,095	1,350
Anger problems	1,00	5,00	1,92	0,85	1,191	0,944
Aggressiveness	1,00	5,00	1,30	0,48	2,372	6,536
Defiant behavior	1,00	5,00	1,45	0,66	2,192	5,841
Locus of control	12,00	22,00	16,91	1,92	0,093	−0,451
Self-control	16,00	52,00	36,69	5,83	−0,257	−0,147

The correlations between locus of control, self-control, internalizing and externalizing problems are presented in Table 3. It can be seen that locus of control and self-control correlate significantly and negatively to each other, in that the higher the external locus of control, the lower the self-control. The correlation of locus of control with each of the internalizing and externalizing problems is positive, in that the greater the external locus of control, the greater the internalizing and externalizing problems. Self-control correlates negatively with each of the internalizing and externalizing problems, in that the greater the self-control, the fewer the internalizing and externalizing problems. Locus of control was most strongly associated with depression, and self-control had the highest correlation with attention problems. Social anxiety showed the lowest correlation with both.

**TABLE 3:** Correlations between locus of control, self-control, internalized and externalized problems in primary.

**Variable**	**1**	**2**	**3**	**4**	**5**	**6**	**7**	**8**	**9**	**10**	**11**	**12**
(1) Locus of control	_											
(2) Self-control	−0,373**	–										
(3) Depression	0,304**	−0,337**	–									
(4) Anxiety	0,192**	−0,231**	0,671**	_								
(5) Social anxiety	0,161**	−0,170**	0,468**	0,535**	–							
(6) Somatic complaints	0,271**	−0,281**	0,649**	0,610**	0,413**	–						
(7) Post-traumatic stress	0,276**	−0,272**	0,680**	0,706**	0,523**	0,634**	–					
(8) Attention problems	0,277**	−0,438**	0,597**	0,543**	0,392**	0,519**	0,524**	–				
(9) Hyperactivity-impulsivity	0,210**	−0,367**	0,490**	0,504**	0,314**	0,457**	0,464**	0,738**	–			
(10) Anger problems	0,208**	−0,330**	0,555**	0,547**	0,368**	0,481**	0,509**	0,574**	0,627**	–		
(11) Aggressiveness	0,169**	−0,285**	0,363**	0,326**	0,179**	0,331**	0,318**	0,439**	0,567**	0,581**	–	
(12) Defiant behavior	0,205**	−0,343**	0,433**	0,357**	0,198**	0,354**	0,338**	0,494**	0,564**	0,544**	0,636**	–

**p < 0.01.

### Secondary School Sample

The values of means, standard deviations, asymmetry, kurtosis, and the possible range of the dimensions of internalizing and externalizing problems of the SENA, locus of control, self-control, and global indexes of secondary SENA are shown in Table 4. It can be seen that almost all asymmetry and kurtosis were also within acceptable ranges of normal distribution (±2) (George and Mallery, 2010; Ryu, 2011). The only exception was antisocial behavior with a kurtosis that was too high. This variable was therefore omitted from further analyses.

**TABLE 4:** Descriptive statistics internalized and externalized problems SENA secondary.

	**Minimum**	**Maximum**	***M***	***DS***	**Asymmetry**	**Kurtosis**
Depression	1	5	2,14	0,85	0,927	0,257
Anxiety	1	5	2,54	0,87	0,588	−0,313
Social anxiety	1	5	2,42	0,82	0,592	−0,030
Somatic complaints	1	5	2,30	0,79	0,728	0,171
Post-traumatic stress	1	5	2,10	0,71	0,819	0,302
Obsessive-compulsive	1	5	2,14	0,74	0,709	0,213
Attention problems	1	5	2,37	0,79	0,610	0,057
Hyperactivity-impulsivity	1	5	2,05	0,68	0,901	0,867
Anger problems	1	5	2,10	0,88	1,010	0,583
Aggressiveness	1	5	1,39	0,50	2,002	5,206
Defiant behavior	1	5	1,60	0,73	1,594	2,610
Antisocial behavior	1	5	1,23	0,36	2,798	10,748
Locus of control	12	24	16,1	2	−0,398	−0,202
Self-control	13	52	35,9	6	−0,18	−0,281

The correlations between locus of control, self-control, internalizing, and externalizing problems of the secondary school students are presented in Table 5. It can be seen that locus of control and self-control also correlate significantly and negatively in this sample.

**TABLE 5:** Correlations between locus of control, self-control internalized and externalized problems in secondary.

	**1**	**2**	**3**	**4**	**5**	**6**	**7**	**8**	**9**	**10**	**11**	**12**	**13**
(1) Locus of control	–												
(2) Self-control	−0,383**	–											
(3) Depression	0,319**	−0,394**	–										
(4) Anxiety	0,189**	−0,313**	0,763**	–									
(5) Social anxiety	0,203**	−0,223**	0,475**	0,553**	–								
(6) Somatic complaints	0,231**	−0,338**	0,708**	0,670**	0,404**	–							
(7) Post-traumatic stress	0,273**	−0,303**	0,737**	0,736**	0,491**	0,634**	–						
(8) Obsessive-compulsive	0,144**	−0,198**	0,523**	0,651**	0,474**	0,488**	0,603**	–					
(9) Attention problems	0,302**	−0,548**	0,510**	0,498**	0,322**	0,504**	0,491**	0,412**	–				
(10) Hyperactivity-impulsivity	0,196**	−0,424**	0,431**	0,463**	0,233**	0,427**	0,464**	0,446**	0,703**	–			
(11) Anger problems	0,212**	−0,392**	0,508**	0,499**	0,223**	0,439**	0,481**	0,391**	0,483**	0,532**	–		
(12) Aggressiveness	0,132**	−0,320**	0,282**	0,232**	0,082**	0,243**	0,280**	0,278**	0,362**	0,476**	0,514**	–	
(13) Defiant behavior	0,190**	−0,374**	0,422**	0,333**	0,127**	0,349**	0,350**	0,271**	0,431**	0,468**	0,512**	0,522**	–

**p < 0.01.

The correlation of locus of control with each of the internalizing and externalizing problems again is positive, so the higher the external locus of control, the higher the internalizing or externalizing problems. In turn, self-control again correlates negatively with each of the internalizing and externalizing problems, so the greater the self-control, the fewer the internalizing problems.

The variable that had the strongest relationship to locus of control was, again, depression. Attention problems also had the highest correlation with self-control, and aggressiveness had the lowest correlation with locus of control and self-control; and the obsessive-compulsive scale had the lowest correlation with self-control.

Prediction of each of the internalizing and externalizing problems using gender, locus of control and self-control is presented in Table 6 for both age groups. It can be seen that, in each regression model, both locus of control and self-control were significant predictors. Only for the aggressiveness dimension did locus of control not function as a significant predictor in the secondary school group, however, locus of control did predict aggressiveness in the primary school group.

**TABLE 6:** Regression models for each internalized and externalized problems in primary and secondary.

**Primary**					**Secondary**				
**Source^a^**	**Predictor**	***R*^2^ model^b^**	**β*^c^***	***p***	**Source^a^**	**Predictor**	***R*^2^ model^b^**	**β*^c^***	***p***
**Internalized problems**									
Depression		0.153			Depression		0.224		
	Self-control		–0.264	0.000		Self-control		–0.321	0.000
	Locus of control		0.208	0.000		Gender		–0.192	0.000
	Gender		–0.050	0.044		Locus of control		0.194	0.000
Anxiety		0.071			Anxiety		0.155		
	Self-control		–0.191	0.000		Self-control		–0.285	0.000
	Locus of control		0.124	0.000		Gender		–0.230	0.000
	Gender		–0.064	0.014		Locus of control		0.077	0.000
Social anxiety		0.051			Social anxiety		0.090		
	Self-control		–0.138	0.000		Self-control		–0.172	0.000
	Gender		–0.115	0.000		Gender		–0.160	0.000
	Locus of control		0.114	0.000		Locus of control		0.136	0.000
Somatic complaints		0.111			Somatic complaints		0.175		
	Self-control		–0.208	0.000		Self-control		–0.295	0.000
	Locus of control		0.194	0.000		Gender		–0.223	0.000
						Locus of control		0.116	0.000
Post-traumatic stress		0.113			Post-traumatic stress		0.148		
	Locus of control		0.203	0.000		Self-control		–0.235	0.000
	Self-control		–0.203	0.000		Gender		–0.168	0.000
	Gender		–0.077	0.003		Locus of control		0.182	0.000
					Obsessive-compulsive		0.046		
						Self-control		–0.168	0.000
						Locus of control		–0.080	0.008
						Gender		–0.054	0.000
**Externalized problems**									
Attention problems		0.206			Attention problems		0.309		
	Self-control		–0.389	0.000		Self-control		–0.506	0.000
	Locus of control		0.132	0.000		Locus of control		0.108	0.000
Hyperactivity-impulsivity		0.158			Hyperactivity-impulsivity		0.182		
	Self-control		–0.324	0.000		Self-control		–0.408	0.000
	Gender		0.137	0.000		Gender		0.045	0.019
	Locus of control		0.082	0.002		Locus of control		0.040	0.049
Anger control problems		0.116			Anger control problems		0.165		
	Self-control		–0.294	0.000		Self-control		–0.366	0.000
	Locus of control		0.099	0.000		Gender		–0.090	0.000
						Locus of control		0.071	0.001
Aggressiveness		0.121			Aggressiveness		0.123		
	Self-control		–0.240	0.000		Self-control		–0.318	0.000
	Gender		0.192	0.000		Gender		0.148	0.009
	Locus of control		0.071	0.009					
Defiant behavior		0.127			Defiant behavior		0.142		
	Self-control		–0.304	0.000		Self-control		–0.353	0.000
	Locus of control		0.088	0.001		Locus of control		0.055	0.009
	Gender		0.067	0.008					

**^*a*^Internalized and externalized problem to be predicted in each model. ^*b*^R^2^ adjusted from the model. ^*c*^β standardized Beta coefficient (β represents the change in the standard deviation of the dependent score resulting from the change of one standard deviation of the independent variable).**

Gender was not a significant predictor in either model. For the primary school sample, gender did not contribute to the model for the prediction of somatic complaints. For depression, the prediction with gender only reached the *p* < 0.05 level. While among the externalizing problems, gender did not predict attention problems nor anger control problems.

In the secondary school sample, gender did not predict attention problems. Likewise, gender goes from third to second place among the predictors in regression models for depression, anxiety, post-traumatic stress, hyperactivity-impulsivity and defiant behavior.

In both samples, for those dependent variables that gender did predict, being female predicted greater internalizing problems, while being male predicts greater externalizing problems. The only exception was anger control problems where being female predicted a higher level.

In the primary school sample, between 5 and 15% of the individual internalizing problems could be predicted by the locus of control, self-control, and gender variables. Social anxiety was least predicted of all independent variables. Depression was the most likely of the internalizing problems to be predicted. Within the same group, 12–21% of the externalizing problems could be predicted by the models. Anger control problems is the least predictable variable, whilst attention problems are most commonly predicted.

In the secondary school sample, locus of control, self-control, and gender predicted 5–22% of the internalizing problems separately, with obsessive-compulsion being the one that could be predicted the least and depression the most, whilst among the externalizing problems the models predicted 12–31%. Aggressiveness problems was the least predictable of all independent variables but attention problems was predicted the most.

## Discussion

The objective of this study was to establish the relationship between locus of control, self-control, internalized and externalized problems in school children from the fourth year of primary education to the last grade of secondary education in northern Chile.

The study results supported our first two hypotheses, showing a significant relationship between both locus of control and self-control and the internalizing and externalizing problems in the expected directions.

Partial evidence was found for hypothesis three that self-control, locus of control, and gender could together significantly predict each of the internalizing and externalizing problems. However, gender did not contribute significantly to the prediction of all of the internalizing and externalizing problems. There were also some variation between the primary school and the secondary school sample groups. In both groups, the regression models better predicted externalizing rather than internalizing problems. In addition, in the secondary sample the models predicted more than in the primary.

Self-control and locus of control were significant predictors in all regression models of internalizing and externalizing problems in both age groups. There was one exception: in the secondary school sample, locus of control did not add anything to the prediction of aggressiveness, although in primary school it was a significant predictor.

The significant negative relationship between self-control and internalizing problems was consistent with previous research, in that higher levels of self-control are negatively associated with symptoms of depression in both primary and secondary schools (Weisz et al., 2004; Jun and Choi, 2013; Nie et al., 2014). Furthermore, the findings for locus of control were also consistent with previous research linking it to internalizing problems such as depression (Culpin et al., 2015; Di Pentima et al., 2019) and anxiety (Król et al., 2019). Thus, both anxiety and depression were significantly associated with locus of control, as both would essentially imply the perception that the individual has no control over his or her life circumstances (Di Pentima et al., 2019).

The present study confirms the importance of considering locus of control and self-control simultaneously in regard to internalizing problems (especially anxiety) as suggested by Ollendick and Grills (2016). Both the association between self-control and externalizing problems (Franken et al., 2015), and between locus of control and externalizing problems are consistent with previous research (Liu et al., 2000).

These results provide evidence for the prediction models proposed in this study in which self-control, locus of control, and gender together, seem to function as predictors of internalizing and externalizing problems in school aged children. The indication of a predictive role was higher for the secondary school group, and it might be explained by the higher demands in school for this age group (Broeren and Muris, 2009; Nie et al., 2014). In Chile, the transition from eighth to ninth grade is an important milestone, as it is in the latter, that final average grades begin to count as part of the score for future university selection. This can generate greater stress than in the early school years, given the greater performance pressure, among other things, reflected in an increase of students that have to retake a year (Ministerio de Educación de Chile, Centro de Estudios, Unidad de Estadísticas, 2018).

Future research should consider other variables that could add more predictive power to these models. For example, psychosocial variables, such as social support and resilience, should be investigated as they can be incorporated into intervention programs (Carle and Chassin, 2004; Attar-Schwartz et al., 2019).

The fact that depression had the highest association with both locus of control and self-control in both age groups, implied that depression is mostly influenced by the ability for, and experience of, control between internalizing problems. Furthermore, although anxiety is usually studied as often as depression, the results of the present study showed that in primary school, both post-traumatic stress and somatic complaints were influenced more by self-control. In secondary school, although anxiety increases, it is still slightly below the last two. Social anxiety increases almost twice as much, although it did not reach the level of general anxiety. These results suggest that it will be necessary to analyze them as separate variables, as well as to assess them together, but as independent sub-dimensions. Studying depression and anxiety from a single score may hide the fact that depression could be more influenced by locus of control and self-control variables.

Between the externalizing problems, it was noteworthy that attention problems were explained mostly by independent variables followed by hyperactivity-impulsivity, although it is evident that the externalizing problems had a stronger relationship to the control variables compared to the internalizing. This is consistent with the importance given to the role of locus of control and self-control in the literature about externalizing problems (Jenson et al., 2011).

It is remarkable that self-control weighs more than locus of control in the prediction of each of the internalizing and externalizing problems. This may be because children and adolescents’ ability to control impulses, emotions, thoughts and behaviors may be more directly related to their mental health than their assessment of whether or not their own behaviors might affect their outcomes. However, the latter can provide information on self-management.

It has been proposed that the hormonal changes at puberty may cause girls to experience greater internalizing problems, without ignoring gender identity and other relevant psychosocial aspects (Sanborn and Hayward, 2003; Natsuaki et al., 2015). All these aspects could help explain the differences between primary and secondary samples concerning gender.

Although the data provided by this research are significant, they have certain limitations, such as its cross-sectional nature, the lack of random selection, and the restrictions inherent in the use of self-report inventories as the primary data source. Since this is a cross-sectional study, it was not possible to establish the direction of the relationship between the variables. Finally, the two versions of the SENA are not directly comparable as they vary in the number and content of questions because they were adapted to each age group.

Despite these limitations, results suggest that both locus of control and self-control could form valuable interventions in the classroom, through the implementation of socio-emotional development programs, such as cooperative play programs and emotional intelligence, which can promote the development of social and emotional competencies that may influence control functions (Garaigordobil et al., 2017). A recent review of self-regulatory interventions suggests that they are effective in children and adolescents to improve their mental health, social skills, behavioral problems and performance (Pandey et al., 2018). Currently, Chile promotes mental health in the school environment with various programs such as Skills for Life (Lara et al., 2012; Murphy et al., 2015), which could integrate work with increasing self-control and internal locus of control to improve the effectiveness of mental health interventions.

While classroom-based interventions may contribute to improved mental health outcomes, it is essential to consider broader interventions that can involve the community and family, since internalizing problems are not limited to school settings (Kang and You, 2018). It has been found that parental supervision, whilst children and adolescents are at home, is a determining factor in the development of an internal locus of control, where a warm and nurturing environment is essential. Furthermore, it has also been observed that harsh discipline is associated with the development of an external locus of control (Ahlin and Lobo Antunes, 2015). This implies the need to support the family in parenting strategies.

Future research, of a longitudinal nature, could address the possible variation in time, both in locus of control and self-control and its relationship with internalizing and externalizing problems, which would support the findings of Sullivan et al. (2017) in relation to locus of control and its effect on depression, and expanding them to include other internalizing problems and self-control. It would be desirable to use random selection and include other regions of Central and Southern Chile, which could better investigate how minority groups score on the variables. Evaluating interventions that are aimed at increasing internal locus of control and self-control are essential in the promotion of mental health of young people.

## Conclusion

Both locus of control and self-control showed a relationship with each dimension of internalizing and externalizing problems, with depression having the strongest association among the internalizing problems, and attention among the externalizing problems. The study results suggest that both self-control and locus of control might have an influence on internalizing and externalizing problems and we therefore recommend them to be included in mental health interventions for children and adolescents both inside and outside the school context.

## Data Availability Statement

The raw data supporting the conclusions of this article will be made available by the authors, without undue reservation, to any qualified researcher.

## Ethics Statement

The studies involving human participants were reviewed and approved by Comité Ético Científico de la Universidad de Tarapacá. Written informed consent to participate in this study was provided by the participants’ legal guardian/next of kin.

## Author Contributions

JF: conception and design of research, preparation of the introduction of the manuscript, data collection, data analysis, and discussion of the manuscript. AC-U: conception and design of research, preparation of the introduction of the manuscript, and discussion of the manuscript. CR: preparation of the introduction of the manuscript, data collection, and discussion of the manuscript. GA and JC: preparation of the introduction of the manuscript and data collection. All authors contributed to the article and approved the submitted version.

## Conflict of Interest

The authors declare that the research was conducted in the absence of any commercial or financial relationships that could be construed as a potential conflict of interest.
